# Consequences of impaired 1-MDa TIC complex assembly for the abundance and composition of chloroplast high-molecular mass protein complexes

**DOI:** 10.1371/journal.pone.0213364

**Published:** 2019-03-13

**Authors:** Peter Schäfer, Stefan Helm, Daniel Köhler, Birgit Agne, Sacha Baginsky

**Affiliations:** Institute of Biochemistry and Biotechnology, Martin-Luther-University Halle-Wittenberg, Biozentrum, Halle (Saale), Germany; Arizona State University, UNITED STATES

## Abstract

We report a systematic analysis of chloroplast high-molecular mass protein complexes using a combination of native gel electrophoresis and absolute protein quantification by MS^E^. With this experimental setup, we characterized the effect of the *tic56-3* mutation in the 1-MDa inner envelope translocase (TIC) on the assembly of the chloroplast proteome. We show that the *tic56-3* mutation results in a reduction of the 1-MDa TIC complex to approximately 10% of wildtype levels. Hierarchical clustering confirmed the association of malate dehydrogenase (MDH) with an envelope-associated FtsH/FtsHi complex and suggested the association of a glycine-rich protein with the 1-MDa TIC complex. Depletion of this complex leads to a reduction of chloroplast ATPase to approx. 75% of wildtype levels, while the abundance of the FtsH/FtsHi complex is increased to approx. 140% of wildtype. The accumulation of the major photosynthetic complexes is not affected by the mutation, suggesting that *tic56-3* plants can sustain a functional photosynthetic machinery despite a significant reduction of the 1-MDa TIC complex. Together our analysis expands recent efforts to catalogue the native molecular masses of chloroplast proteins and provides information on the consequences of impaired accumulation of the 1-MDa TIC translocase for chloroplast proteome assembly.

## Introduction

Protein/protein interactions determine complex biosynthetic activities such as the synthesis of metabolites, DNA, RNA and proteins. Interactions between proteins can be transient and conditional and depend on the presence of ligands. These protein interactions are difficult to assess with biochemical approaches making a functional *in vivo* or *ex vivo* assay such as bimolecular fluorescence complementation or yeast two hybrid assays necessary. Stable protein interactions allow protein complex analysis and identification by classical biochemical techniques such as native gel electrophoresis or gel permeation chromatography [1, 2]. With the advent of high-throughput cloning and protein identification technologies, large-scale tandem-affinity purification strategies were designed that rely on introducing a tagged protein into the cell and isolating stable interaction partners in association with the protein of interest [3]. Using the above techniques, examples for newly discovered metabolite channeling units -so called metabolons- were reported that include the sporopollenin metabolon in pollen wall formation [4] and a flavonoid-biosynthetic metabolon in Arabidopsis protoplasts [5].

In chloroplasts, several approaches characterized the native protein complex proteome by classical biochemical techniques such as BN PAGE, colorless native (CN-) PAGE, gel permeation chromatography and/or glycerol density gradient centrifugation [6–12]. Apart from the photosynthetic complexes photosystem I and II, the ATPase and the cytochrome b6/f complex, the most-studied chloroplast protein complexes entail the plastid RNA polymerase and the protein import machinery at the outer (TOC) and inner envelope membrane (TIC). The latter complexes are essential for chloroplast biogenesis and resemble highly complex macromolecular assemblies whose complete subunit composition and functional interactions are not yet fully understood [8, 13–15]. Furthermore, there is increasing interest in the composition of chloroplast protease complexes such as the CLP proteases and the FtsH proteases that are involved in the homeostasis of thylakoid membrane proteins and -in case of envelope associated FtsH/FtsHi complexes- in plastid protein import and/or quality control [12, 16–18].

Recent efforts to map the high molecular mass chloroplast proteome suffered from low resolution because there is a tradeoff between resolution and scalability in large-scale mass spectrometric analyses. Consequently, the number of samples is reduced to keep instrument time low to allow the analysis of replicates under a variety of different experimental or biological conditions. For example, Olinares and colleagues used five sample bins for the identification of soluble protein complexes in a molecular mass range from 800 kDa to >5 MDa [8] and quantified proteins by normalized spectral counting (nSpC). The most recent study reported by Lundquist and colleagues [12] used BN-PAGE to dissect protein complexes using six bins to cover the entire accessible molecular mass range up to several MDa. The final bin covered the molecular masses > 669 kDa such comprising the most important high molecular mass protein complexes in a single bin, preventing a differentiated complex assessment.

With the study reported here, we expand these recent efforts by combining BN-PAGE separation of protein complexes with absolute protein quantification by MSE. MSE is a data-independent acquisition method that rapidly switches between MS and MS/MS acquisition modes, thereby providing high-resolution quantitative information for all fragmented precursor ions in a sample. This enables absolute protein quantification by setting the intensity read-out of proteotypic sample peptides in relation to those of an external standard (e.g. 10 fmol of glycogen phosphorylase B). The correlation of peptide signal intensity and protein concentration is used to infer the absolute abundance of proteins in the sample [19–20]. With this experimental setup, every identified protein is characterized by its position in the gel and by its absolute abundance. The obtained numeric values allow hierarchical clustering to identify proteins with similar profiles as potential interaction partners within a protein complex. We used this approach to characterize the effect of the *tic56-3* mutation on the 1-MDa TIC complex and the assembly of the plastid high-molecular mass proteome. The 1-MDa TIC complex consists of four known subunits comprising Tic214 (i.e. YCF1), Tic100, Tic56 and Tic20, and it has been reported as the general protein import pathway across the inner envelope membrane [15]. This view has been challenged so the characterization of the weak mutant *tic56-3* is expected to provide new information about its functional context [21]. Furthermore, the perturbation in the 1-MDa TIC complex adds an additional component to the distance matrix for the hierarchical clustering such increasing the significance of the cluster.

Our analysis provides a robust identification and quantification of the major chloroplast proteins complexes and provides new information on their compositions. Despite a significant reduction in the abundance of the 1-MDa TIC complex in *tic56-3*, we could not identify significant differences in the assembly of protein complexes at the thylakoid membrane, with the ATPase complex as only exception. We could show that a recently identified FtsH/FtsHi complex at the inner envelope membrane accumulates independently of the 1-MDa TIC complex, even though they form a supercomplex during protein translocation in wildtype chloroplasts [18]. We discuss here a selection of protein complexes of interest to us, but there is more to discover in this dataset. To this end, our data are available as a resource for further studies.

## Materials and methods

### Plant material and growth conditions

The following *Arabidopsis thaliana* lines were used: *tic56-3* (FLAG579H12), Ecotype Wassilewskija (Ws) was grown as wild-type control for the *tic56-3* wildtype comparison. Plants were grown under short-day conditions (8 h/d light at 21°C) on 1% (w/v) agar plates containing 0.5 × concentrated Murashige and Skoog (MS) medium supplemented with 0.8% (w/v) sucrose.

### Blue-native polyacrylamide-electrophoresis and in-gel tryptic digest

Chloroplasts were isolated by Percoll density gradient centrifugation as described previously and washed three times to remove residual Percoll contaminations [22]. After the final washing, the chloroplast pellet was resuspended in 50 mM BisTris/HCl (pH 7.0), 0.5 M 6-amino-caproic acid, 10% glycerol (v/v), 1% digitonin (w/v) and supplemented with a protease inhibitor cocktail (0.1% (v/v), Sigma-Aldrich) and incubated for 20 minutes under gentle shaking. The sample was subsequently centrifuged for 10 minutes at 100.000xg and the supernatant used for further analyses. All steps were carried out at 4°C. BN-PAGE was carried out essentially as described by Kikuchi and colleagues [14]. In short, solubilized chloroplast proteins were mixed with Coomassie G250 in a digitonin/Coomassie ratio of 8:1 and 200 μg protein per lane was loaded onto a 5–15% acrylamide/bisacrylamide (37.5:1)-gradient gel comprising 50 mM BisTris/HCl (pH 7.0), 0.5 M 6-amino-caproic acid, and a glycerol gradient from 5–9%. Gel electrophoresis was carried out at 4°C with a cathode buffer consisting of 50 mM Tricin, 15 mM BisTris, 0.02% (w/v) Coomassie-G250 that was replaced after approximately 75% of the running distance by the same buffer without Coomassie-250. The anode buffer contained 50 mM BisTris/HCl (pH 7.0). After electrophoresis, every gel lane was cut into nine (the first two replicates) or seven (replicate three and four) equally sized slices (0.5 cm) in the molecular mass region between 600 kDa and approximately 4-MDa and every gel piece was subjected to in-gel tryptic digest as described previously [23]. Tryptic peptides were dried and stored at -80°C until further analysis.

### Protein identification and quantification by HD-MSE mass spectrometry

Dried peptide pellets were dissolved in 2% (v/v) ACN and 0.1% (v/v) FA and injected into an ACQUITY UPLC System connected to a Synapt G2-S mass spectrometer (Waters, Eschborn, Germany). Nano-LC separation (140 min gradient) and HD-MSE data acquisition was performed as described previously [20, 24]. Data analysis and interpretation was carried out by ProteinLynx Global Server (PLGS 3.0.1, Apex3D algorithm v. 2.128.5.0, 64 bit, Waters, Eschborn, Germany) with automated determination of chromatographic peak width as well as MS TOF resolution. Lock mass value for charge state 2 was defined as 785.8426 Da/e with a lock mass window set to 0.25 Da. Low/high energy threshold was set to 180/15 counts, respectively. Elution start time was at 5 min, intensity threshold was set to 750 counts. Databank search query was performed with Protein Lynx Global Server (PLGS version 2.5) and was carried out as follows: Peptide and fragment tolerances was set to automatic, two fragment ion matches per peptide, five fragment ions for protein identification, and two peptides per protein were required for protein identification. Primary digest reagent was trypsin with one missed cleavage allowed. According to the digestion protocol, carbamidomethyl on Cys was set as fixed and oxidation on Met as variable modification. The false discovery rate (FDR) was set to 4% at the protein level. MSE data were searched against the modified *A*. *thaliana* database (TAIR10, ftp://ftp.arabidopsis.org) containing common contaminants (ftp://ftp.thegpm.org/fasta/cRAP/crap.fasta) and rabbit glycogen phosphorylase B (P00489) was used as internal quantification standard [24]. Protein quantification was performed by correlating the signal intensity of the three best ionizing peptides of every identified proteins with those of the spiked standard, i.e. 10-fmol rabbit glycogen phosphorylase B. The mass spectrometry proteomics data have been deposited to the ProteomeXchange Consortium (http://proteomecentral.proteomexchange.org) via the PRIDE partner repository [25]. To assess regulation of protein abundances, protein quantities were normalized to ppm-values where indicated. To this end, quantities per data set were summed and set as one million. Each protein quantity was proportionated as a part of its data set to obtain its ppm value. Hierarchical clustering was performed with Multi Experiment Viewer using Pearson correlation as distance metric and average linkage clustering as linkage method. We kept the sample tree order constant and allowed optimization of gene leaf order (http://mev.tm4.org).

### Antibodies

Antibodies against atTic20-I (At1g04940) were described previously [26].

## Results and discussion

### Isolation of native complexes and quantification by MS^E^ mass spectrometry

**Fig 1:**
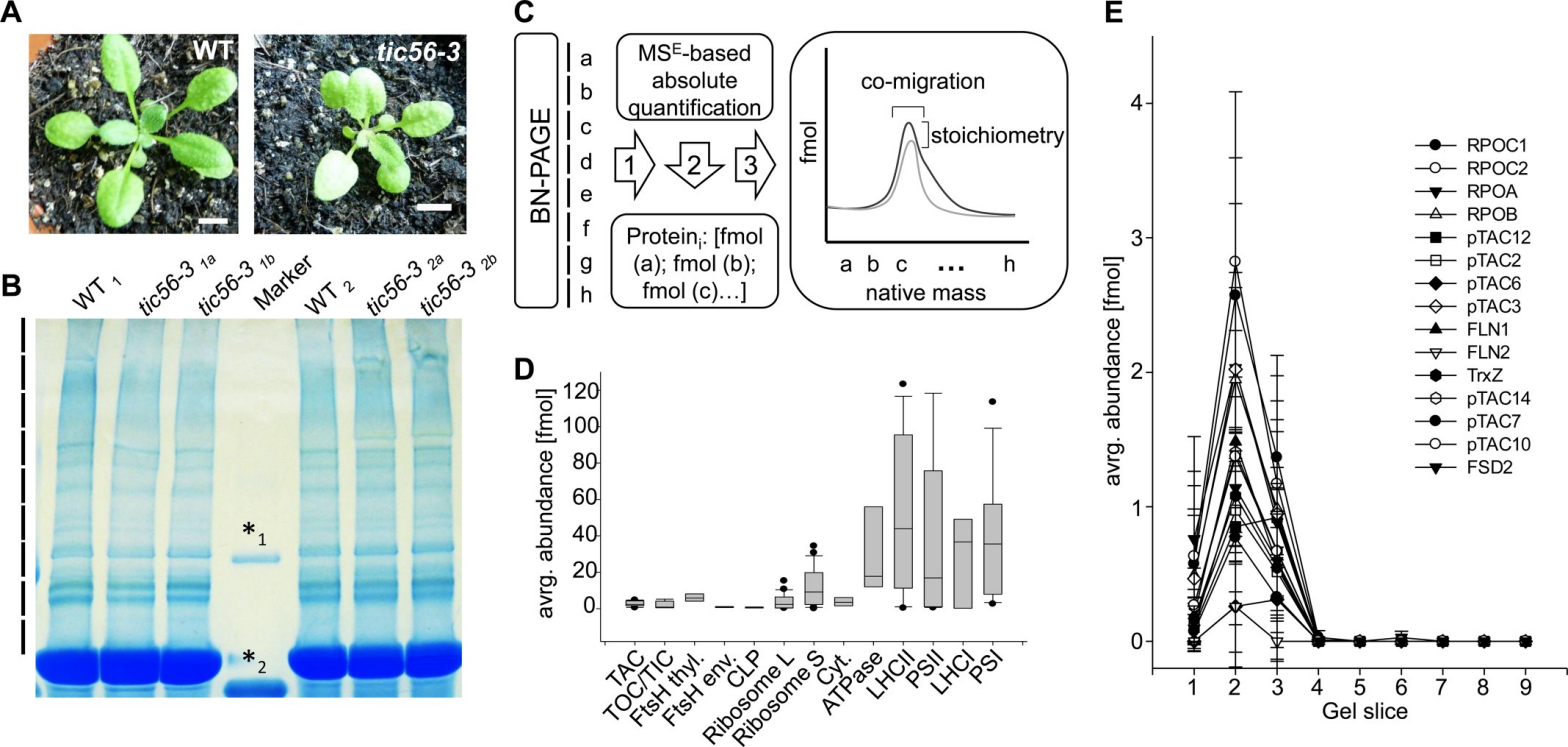
Comparison of the high molecular mass proteome of wildtype and *tic56-3* mutant chloroplasts by BN PAGE. (A) Four-week old plants from *Arabidopsis thaliana* Ecotype Ws. and the *tic56-3* mutant were used for the analysis. The bar represents 1 cm. (B) Extracted chloroplasts were solubilized in 1% digitonin (w/v) and subjected to BN PAGE. The region above the RubisC/O band were cut into nine (replicates 1 and 2) and seven (replicates 3 and 4) equally sized slices. The following markers are displayed, *1: 440kDa, *2: 880kDa (C) Schematic workflow for the BN-PAGE analysis and the quantification of protein complexes. The result is a 2-dimensional matrix in which every identified protein is represented by two values, a fmol abundance value and the position in the gel, i.e. gel slice (a, b, c…h). Based on co-migration and stoichiometry, proteins can be assigned to complexes. (D) Box plot representation of subunit abundance distribution for the major chloroplast protein complexes. The data were extracted from four wildtype replicates. (E) Migration and abundance profile of the plastid transcriptionally chromosome (pTAC). Standard deviations were calculated from four biological replicates.

We isolated chloroplasts from wildtype and *tic56-3* mutants and solubilized 200 μg chloroplast proteins prior to BN-PAGE in a 1% digitonin-containing buffer (Fig 1A and 1B). Using solubilized chloroplast proteins, we performed electrophoretic separation on a 4–15% BN-PAGE at 4°C overnight (Fig 1B). Extrapolating from a regression curve, the gel allowed separation of protein complexes up to around 4 MDa, which is similar to previous studies where gel-filtration was used for native complex isolation from chloroplasts [8]. We cut the gel in the region between 600 kDa and approximately 4 MDa into nine equally sized slices and identified and quantified the protein constituents of every gel slice by HD-MS^E^ mass spectrometry, as previously described (Fig 1C) [20]. Altogether, 424 proteins were identified and quantified after removal of very low abundance proteins (< 1 fmol sum in all fractions) that would dilute the cluster with low confidence associations (S1 Table). The experimental design provides high-resolution separation of protein complexes in the molecular mass dimension (gel slice) and in the abundance dimension where we obtained *fmol* values for every protein in every gel slice (Fig 1C–1E).

We first assessed the abundance of protein complexes in chloroplasts using the wildtype data as a reference (Fig 1D). The median abundance of complex subunits is displayed as a box plot. The least abundant complexes are the CLP complex and the envelope FtsH/FtsHi complexes, followed by the TOC/TIC complexes and the *plastid transcriptionally active chromosome* (pTAC) (Fig 1D). The thylakoid FtsH complexes, the ribosomes and the cytochrome b_6_f complex are of intermediate abundance. Protein complexes with the highest abundance are the thylakoid membrane complexes ATPase, LHCII, PSII, LHCI and PSI. The abundance distribution of individual subunits of the depicted complexes (i.e. the width of the boxes) can be taken as a quality indicator for the solubilization efficiency and the stability of the complexes in BN PAGE. As can be seen in Fig 1D, the distribution is narrow for the low and intermediate abundance complexes while it is high for the small subunit of the ribosome and the photosynthetic complexes. It is unlikely that this distribution reflects subunit stoichiometry of the complexes. Rather, it can be attributed to protein complex dissociation throughout BN PAGE analysis and/or incomplete solubilization of hydrophobic proteins (Fig 1D).

The distribution and migration profiles of proteins within a complex is exemplified for the pTAC complex in Fig 1E and in S1 Fig. The abundance profile is calculated with standard deviations from four biological replicates. Most subunits of the transcriptional core of pTAC co-migrate in a sharp peak of approx. 3 MDa (Gel slice 2) while only pTAC6 (At1g21600) and pTAC12 (At2g34640) have an additional maximum at approx. 2 MDa (Fig 1E). The plastid encoded RpoC1 (AtCg00180) and RpoC2 (AtCg00170) subunits have the highest abundance within the pTAC complex (2.7–2.9 fmol), followed by RpoA (AtCg00740), RpoB (AtCg00190) and pTAC3 (At3g04260) (approx. 2 fmol). The abundance of the remaining subunits ranges from 0.8 fmol to 1.4 fmol, suggesting a subunit stoichiometry between two or three to one. FLN2 (At1g69200) and pTAC6 (At1g21600) are not considered for the stoichiometry assessment because their abundance is much lower, i.e. approx. 0.2 fmol. The subunit composition depicted here reflects the transcriptional core of the pTAC [27], which forms a complex that appears stable under the chosen detergent conditions.

### Hierarchical clustering reveals protein complex composition

**Fig 2:**
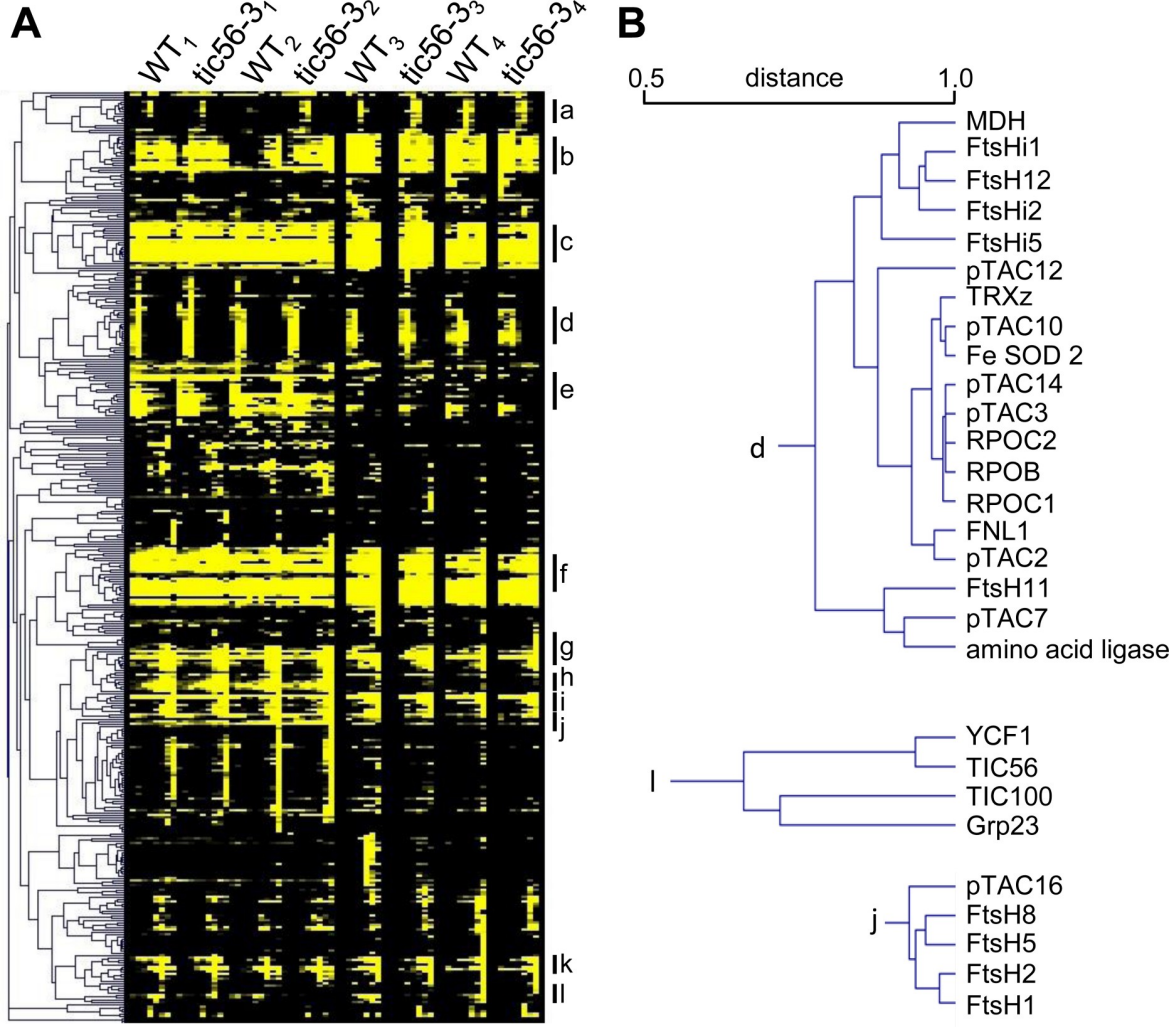
Hierarchical cluster from absolute protein quantification data (HD-MSE) of BN PAGE resolved protein complexes. (A) Complete cluster built from 424 protein identifications. We used the 2-dimensional data on migration and abundance for every identified protein in four biological replicates in wildtype and in the *tic56-3* mutant. The main cluster comprise: a. NADH dehydrogenase, b. ribosome small subunit, c. photosystem II (PSII) with light harvesting complex II (LHCII), d. pTAC with envelope FtsH/FtsHi complex, e. ribosome large subunit, f. PSI with LHCI, g. ATPase, h. thylakoid FtsH proteases, i. RubisCO, j. glycerinaldehyd 3-phosphate dehydrogenase (GAP-DH), k. ferritin, l. 1-MDa TIC complex. (B) Detailed view of three extracted cluster. The distance metric (from Pearson correlation) is provided in the panel above the dendrogram branches.

With the two-dimensional matrix of position and abundance, we performed an unbiased analysis of protein co-migration by hierarchical clustering with data from four biological replicates for the wildtype vs. *tic56-3* comparison. A tight clustering is observed for the subunits of the thylakoid membrane complexes PSI, PSII, the ATPase (α, β, γ, δ and ε subunits and the F0 beta subunit) and the thylakoid-associated FtsH proteases (S2 Fig). Prevailing soluble complexes comprise the large and the small ribosomal subunits, a ferritin complex consisting of ferritin 1, 2 and 4 and the pTAC (S2 Fig, Fig 2A). The large and the small subunits of the ribosome cluster in two main cluster comprising eleven large and 14 small subunit proteins. Notably, chloroplast stem loop-binding protein CSP41-a (At3g63140, in S2 Fig designated as *chloroplast stem-loop binding protein of 41 kDa*) and two RNA-binding proteins (At1g09340 –designated as *chloroplast RNA binding*, At2g35410 –designated as *RNA-binding (RRM/RBD/RNP motifs) family protein*) form a small sub-cluster that is associated with the cluster of the small subunits of the ribosome (Fig 2A, S2 Fig). The wide distribution of abundances of the individual ribosomal subunits in the small and the large subunit can be attributed to incomplete stability throughout the separation, which has also been observed in a detergent–free stroma preparation after gel filtration [8]. Compared to this study, the ribosomal complexes are comparatively more stable in our hands, even in the presence of 1% digitonin (S2 Fig). BN-PAGE was originally developed for the characterization of integral membrane protein complexes. We identified photosystem I and II along with their light harvesting proteins. We find two proteins implicated in the regulation of thylakoid architecture (CURT1A - At4g01150 and CURT1C - At1g52220) to cluster with PSI subunits, similar to observations made in previous studies [28].

Arabidopsis chloroplasts contain nine FtsH proteases and five presumably inactive enzymes that lack the consensus Zn^2+^-binding motif in the M41 peptidase domain. These have been named FtsHi to indicate their lack of proteolytic activity (“i”–inactive). In chloroplasts, hetero-oligomeric complexes that are involved in the assembly maintenance of a functional thylakoid membrane system have been described [17, 29–31]. The hetero-oligomeric complexes comprise subunits of the A-type and of the B-type FtsHs that derived from gene duplication [32]. We identified all four major thylakoid associated FtsHs at concentrations of approx. 4 fmol (FtsH1 and FtsH8) and approx. 8 fmol (FtsH2 and FtsH5) thus confirming estimates about a one to two ratio between A- and B-type FtsH proteases at the thylakoid membrane system [29]. The cluster suggests that FtsH8 and FtsH5 on the one hand and FtsH1 and FtsH2 form hetero-hexameric complexes in a ratio of two to four, respectively (Fig 2B).

In addition to the thylakoid FtsH proteases, several FtsH and FtsHi enzymes localize to the envelope membrane system. Our data clearly separate the envelope from the thylakoid FtsH enzymes by their molecular mass and their abundance. While the envelope proteases form very large protein complexes with molecular masses of around 2 MDa, the thylakoid FtsHs are smaller and accumulate to higher abundance [18] (S1 Table, S2 Fig). The envelope protease FtsH12 clusters most closely with FtsHi1, FtsHi2, MDH (AT3G47520) and FtsHi5 (Fig 2) supporting a recent report that the FtsHi enzymes form hetero-oligomeric complexes with FtsH12 and MDH, respectively [18]. Recent data furthermore suggested that MDH and FtsH12 are functionally associated and essential for chloroplast biogenesis [18]. It is thus very likely that the FtsH/FtsHi complex at the envelope membrane is a hetero-oligomeric complex that comprises MDH. Intriguingly, YCF2 was recently reported to associate with this complex [18]; however, we could not identify this protein in the BN-PAGE slices for reasons currently unknown to us (S1 Table).

We identified several known members of the translocon at the outer chloroplast envelope membrane (TOC), including Toc159, Toc33, Toc34 and Toc75. These proteins form complexes during the import process but the complex is unstable under the BN PAGE conditions reported here. In contrast, the recently identified 1-MDa translocon at the inner envelope membrane forms a stable complex that is identified by the unsupervised hierarchical cluster (Fig 2B). In addition to the known subunits Tic214 (i.e. YCF1), Tic100 and Tic56, an unknown protein (Grp23) clusters with the other members of the 1-MDa Tic complex suggesting that it associates with the complex. Tic20-I (At1g04940) was never identified by mass spectrometry and we do not detect it here.

### Identification and quantification of complexes in wildtype and tic56-3 mutant plastids

**Fig 3:**
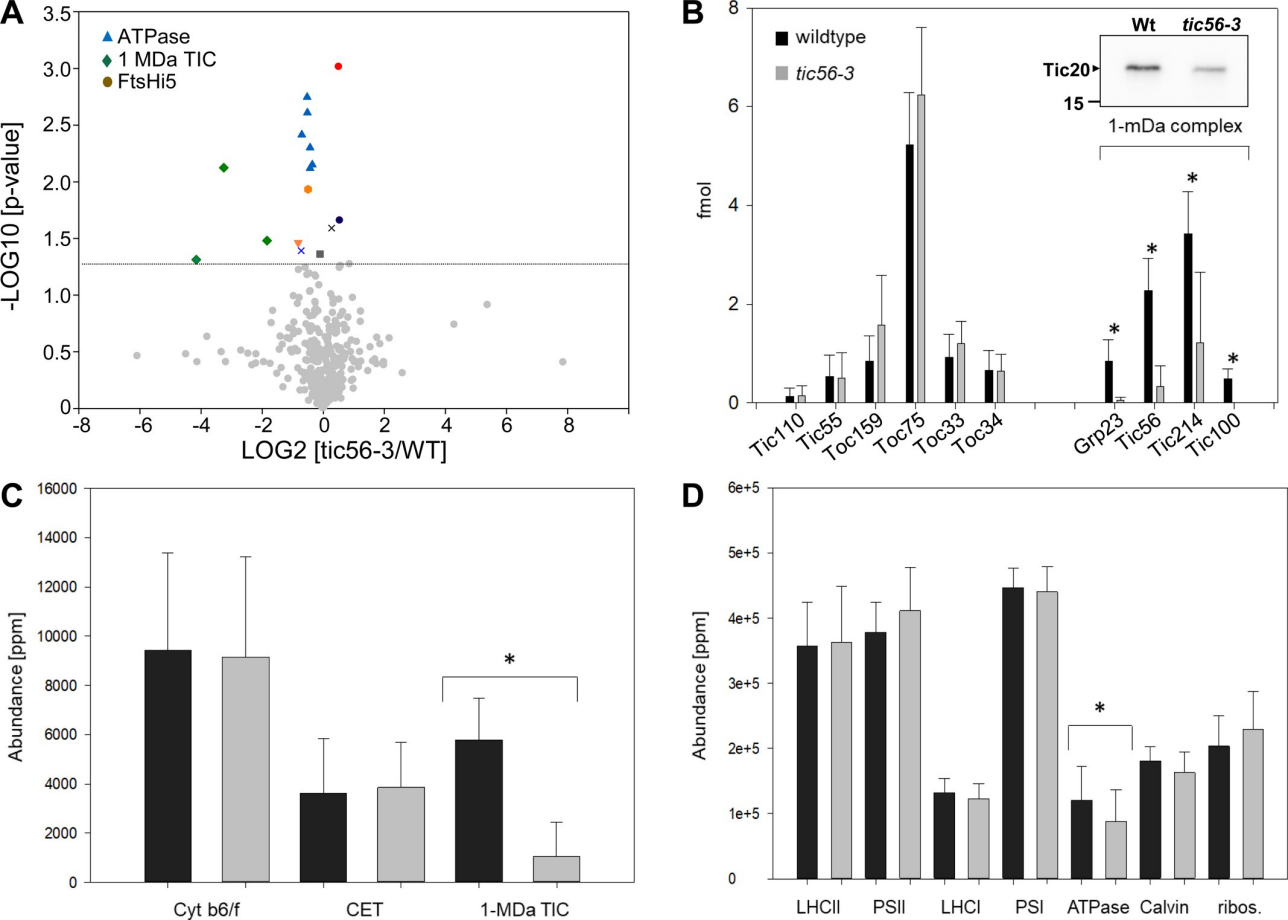
Quantitative proteome analysis and comparison between wildtype and *tic56-3*. (A) To compare the abundance of proteins in wildtype and the *tic56-3* mutant, we calculated the total abundance of every identified protein from the sum of its abundance in the individual gel slices. These data were normalized and transformed into ppm values [20]. We then calculated average Log2 ratios for every protein from its abundance in wildtype and *tic56-3* mutant plastids and calculated a p-value from a two-sided t-test from the four replicates (two-sided paired t-test). Proteins with a significantly changed abundance in *tic56-3* compared to wildtype are labeled in color (above the line indicating a p-value of 0.05). (B) Comparison of TOC and TIC protein abundances in wildtype and the *tic56-3* mutant illustrates the specificity of the 1-MDa TIC complex downregulation. Tic20-I (see inset from an immunoblotting experiment), Grp23, Tic56, Tic214 and Tic100 have a significantly decreased abundance in the *tic56-3* mutant, while all other translocon components, i.e. Tic110, Tic55, Toc159, Toc75, Toc33 and Toc34 are unaffected. The error bars illustrate the standard deviation from four replicates. (C) All identified protein were classified into MapMan bins [33]. The ppm values for every identified protein in every bin were summed up to give rise to the total amount of ppm in every bin. We display here the data for the bin cytochrome b6/f complex, proteins involved in cyclic electron flow (CET) and the 1-MDa TIC complex including Grp23. (D) Same as in C except that the abundance of proteins for the photosynthetic complexes, the Calvin cycle and the ribosome are displayed. Significance (p<0.05) was calculated from a two-sided pairwise t-test and is illustrated with a “*”.

We next compared the abundances of proteins and protein complexes between wildtype and *tic56-3* plastids. To this end, we summed up the fmol values from every gel slice to result in a single fmol value for every protein. Based on four replicates, we calculated p-values from a two-sided t-test to test for the rejection of the null hypothesis, i.e. that the protein abundances are identical in wildtype and the mutant (S2 Table). The volcano plot presented in Fig 3A revealed 17 proteins with significant differences between wildtype and *tic56-3* plastids (two-sided paired t-test, p<0.05), among them six subunits of the chloroplast ATPase, three subunits of the 1-MDa TIC complex, Grp23 (At2g43630), that clusters with the 1-MDa complex (Fig 2B), carbonic anhydrase (At3g01500), fibrillin (At1g51110), PsaK (At1g30380) and FNR1 (At5g66190). The abundance of these proteins is significantly reduced in *tic56-3* compared to wildtype. Significantly higher protein abundance in *tic56-3* was observed for FtsHi5, Rubisco activase and the unknown protein At4g13500 (Fig 3A). We plotted the individual components of the import machinery in Fig 3B to visualize the effect of the *tic56-3* mutation. These data show that the TOC members, Tic55 and Tic110 are not changed in the *tic56-3* mutant while the members of the 1-MDa TIC complex are significantly decreased in abundance, including Tic20-I (see inset) and Grp23. Thus, the strongest effect of the *tic56-3* mutation on the plastid proteome is the downregulation of the 1-MDa TIC complex and of ATPase. None of the photosynthetic complexes at the thylakoid membrane is significantly affected in the *tic56-3* mutant (Fig 3C and 3D), consistent with the staining intensities of the major complexes after BN PAGE (Fig 1B). Note that we made the complex abundance comparison on ppm-normalized data (Fig 3 A, 3C and 3D).

**Fig 4:**
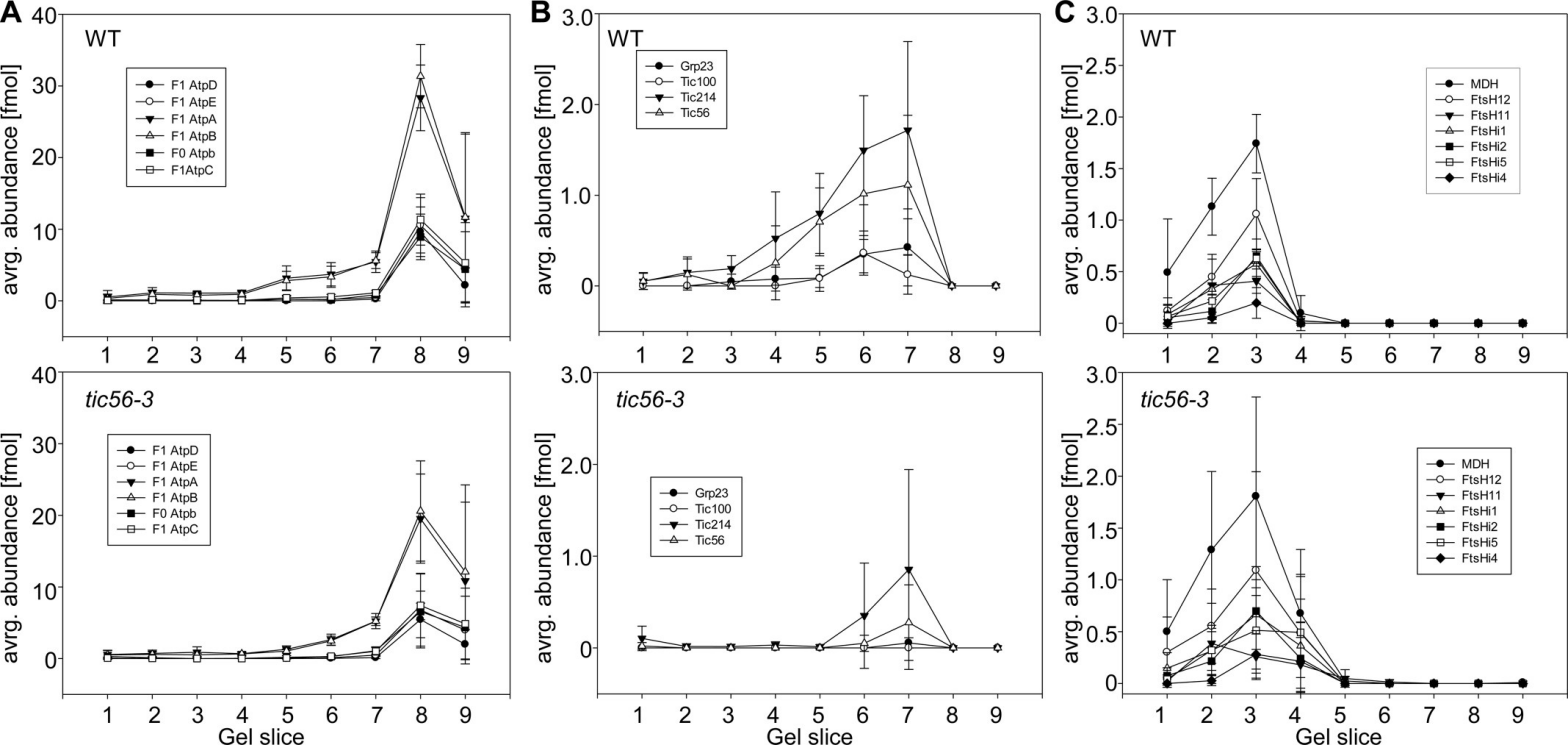
Migration profiles of chloroplast protein complexes in wildtype and in *tic56-3* plastids. Displayed are the migration profiles for ATPase (A), the 1 MDa TIC complex (B) and the FtsH/FtsHi complex (C) in wildtype (upper panel) and in *tic56-3* (lower panel), respectively. We aligned the replicates 1 and 2 with the replicates 3 and 4 by the maximum abundance of the displayed proteins. Provided are the standard deviations from protein abundance values obtained in four biological replicates.

We visualized the migration pattern of chloroplast ATPase, the 1-MDa TIC complex and the envelope FtsH/FtsHi complex in BN PAGE data from wildtype and *tic56-3* mutants (Fig 4). ATPase was identified with five of its F1 and one of its F0 subunits in wildtype and in *tic56-3* (Fig 4A). The subunit stoichiometry for the F1 part is correctly reproduced in BN PAGE with three α and β subunits and one γ, δ and ε subunit forming the complex (Fig 4A). In the *tic56-3* mutant, the subunit stoichiometry is identical, but all subunits have lower abundance compared to wildtype (see fmol on the y-axis Fig 4A). Similarly, the abundance of the 1-MDa TIC complex is significantly reduced. Tic214 (i.e. YCF1) is still detectable along with minor amounts of Tic56 (Fig 4B), while Tic100 and Grp23 are below the detection limit. For the FtsH/FtsHi complex at the envelope, we find FtsH12, FtsHi1, FtsHi2, FtsHi5, FtsHi4 and MDH to co-migrate at around 2-MDa, while FtsH11 seems to form slightly larger complexes (Fig 4C). This is consistent with the clustering that separated FtsH11 from the other FtsH/FtsHi, and with recent data on the subunit composition of this complex [18]. For this complex, we find slightly increased abundance in the *tic56-3* mutant compared to wildtype, which is significant for FtsHi5 (Fig 4C and Fig 3A). In a paired t-test (two-sided paired t-test) with all subunits of this complex, we find an overall significantly higher abundance (p-value 0.0001) in *tic56-3* compared to wildtype even though the standard deviations for the *tic56-3* measurements are high. The higher abundance could be interpreted as an upregulation to compensate for the downregulation of the 1-MDa TIC complex, which supports a function of the FtsH/FtsHi complex in protein import [18]. However, the FtsH/FtsHi complex can accumulate independently of the 1-MDa TIC complex, even though both form complexes during protein translocation in wildtype [18]. This suggests that the FtsH/FtsHi complex can function independently of the 1-MDa TIC complex and sustain efficient import in fully developed chloroplasts.

In conclusion, we report here a detailed analysis of chloroplast protein complexes by BN PAGE. We found 1% digitonin (w/v) suitable for solubilization of chloroplast proteins, but other detergents or combinations thereof may as well be suitable for this purpose and deliver partially complementary results [34]. While Olinares and colleagues used soluble stroma preparations lacking any detergent for gel permeation chromatography, Lundquist and colleagues used n-dodecyl-ß-D-maltoside (DDM) in a concentration of 0.5% (w/v) in BN PAGE. With both detergents, i.e. DM and digitonin, high molecular mass protein complexes are preserved and accessible for a detailed subunit analysis. We cannot exclude, however, that very hydrophobic proteins are neglected and their abundance underestimated under the solubilization conditions reported here. This must be taken into account when assessing absolute protein abundances, but it is less relevant for the comparison between wildtype and *tic56-3*. Since many protein complexes were identified at their intact molecular mass (e.g. the 1-MDa TIC complex and the 2 MDa FtsH/FtsHi complex) and an almost stoichiometric abundance distribution (ATPase), we think that solubilization efficiency is not a systematic problem.

Nonetheless, despite the overall good data quality several other critical points require attention. First, the fmol values we report are only predictive for subunit stoichiometry in the case of stable protein complexes. Complexes that dissociate during BN PAGE may give rise to inaccurate quantification results that are manifested as far spreading of protein abundances in protein complexes. This was observed for the photosynthetic complexes and the ribosomes (see e.g. box plot in Fig 1D). In contrast, the data for stable protein complexes such as the transcriptional core of the pTAC have sufficient robustness to allow an assessment of subunit stoichiometry. Another problematic issue concerns the absolute quantification of highly abundant proteins such as the photosynthetic complexes. Apart from potential inaccuracies because of incomplete solubilization, we have recently shown that the absolute quantification strategy employed here underestimates high abundance proteins, which is due to ion mobility separation (IMS) that was used for the MSE measurement. The latter is known to results in rapid detector saturation preventing highly abundant proteins from being reliably quantified [20]. Thus, we conclude that the dynamic range of complex abundances is higher than suggested by our data (e.g. box plot in Fig 1D).

The data presented here expand recent efforts to catalogue the oligomeric state of chloroplast proteins by increased resolution and robust absolute quantification. Similar to the published information, we identify many proteins in high molecular mass fractions. Our data allow hierarchical clustering to assess complex composition since the resolution of the migration pattern is high and the *tic56-3* mutation serves as additional distance criterion to identify complex associations. The hierarchical cluster suggests complexes that are consistent with published literature; e.g. it confirms MDH association with an FtsH/FtsHi complex at the envelope membrane [18] certifying high data quality.

The quantification of protein complexes from *tic56-3* chloroplasts revealed a significant downregulation of the 1-MDa TIC complex including the translocation channel Tic20-I (Fig 3B) [35]. Tic214 and Tic56 are still detectable in the 1-MDa region while Tic100 and the associated Grp23 (Fig 2B) are below the detection limit. For Tic214, an additional function in the assembly of photosystem I, cytochrome b6/f complex and NADH dehydrogenase has been reported [36] and it is therefore likely, that it has functions independent of the 1-MDa TIC complex. Similarly, Tic56 plays a role in chloroplast ribosome assembly [37]. The downregulation of the 1-MDa TIC is accompanied by a downregulation of chloroplast ATPase and a slight but significant upregulation of an envelope-associated FtsH/FtsHi complex (Fig 4) that was identified by hierarchical clustering and co-migration (Fig 2B and Fig 4). The functional connections between these observations is unknown, but recent data suggested that FtsH/FtsHi proteases are involved in chloroplast biogenesis and in protein import regulation [18, 38].

The latter aspect is supported by the association of the FtsH/FtsHi complex with the plastid import machinery [18], that was also observed in the *kinase at the outer chloroplast membrane* (KOC) 1 interaction network [39]. The original report identified the YCF2 protein as a subunit of the FtsH/FtsHi complex that was proposed to form a hexameric structure with FtsHi1, FtsHi2, FtsHi4, FtsHi5 and FtsH12 [18]. For reasons unknown to us, we were unable to detect YCF2 from the BN PAGE slices but it seems likely that this is due to technical limitations of the MSE-optimized search algorithm rather than its absence from the complex. Alternatively, the highly hydrophobic YCF2 protein may not be solubilized with digitonin. FtsH proteases belong to the AAA-family of proteins that form two hexameric ring-like structures in eubacteria that recognize unfolded proteins and degrade them, such constituting a protein quality control system [30, 40]. The complex(es) identified here, however, comprise FtsHi proteins that lack the consensus Zn^2+^-binding motif in the M41 peptidase domain and are thus catalytically inactive [30] with the exception of FtsH12, whose catalytic activity is dispensable for the essential function of this complex in chloroplasts [18]. It is therefore noteworthy that mitochondrial AAA-proteases do not require proteolytic activity to mediate protein translocation across the inner membrane [41–42].

## Supporting information

S1 Fig BN-PAGE profiles of individual pTAC subunits in fmol (y-axis) in the BN-PAGE gel slices (x-axis) (see also Fig 1E in the main text). Note that the data are not normalized.
(PPTX)

S2 Fig Hierachical cluster obtained with the two-dimensional data matrix. (TIF)

S3 Fig BN-PAGE migration profiles of subunits of the FtsH/FtsHi/MDH complex from wildtype (see also Fig 4C). The BN-PAGE gel slice number is provided on the x-axis, the abundance (not normalized, in fmol) is provided on the y-axis.
(PPTX)

S1 Table Identification and quantification of proteins from gel slices (1–9) in four replicates from wildtype and tic56-3 plastids. Presented data are fmol values that were determined by compariosn to 10 fmol glycogen phosphorylase b as previously described [19–20].
(XLSX)

S2 Table Normalization of total fmol abundances to ppm values, calculated for all four replicaes from wildtype and tic56-3 plastids. The paired t-test was performed on ppm-normalized data.
(XLSX)
