# Supplementary material for: Consequences of impaired 1-MDa TIC complex assembly for the abundance and composition of chloroplast high-molecular mass protein complexes
Source: PLoS One. 2019 Mar 13;14(3):e0213364. doi: 10.1371/journal.pone.0213364 (PMC6415892; doi:10.1371/journal.pone.0213364)
Supplement: S1 Fig — Note that the data are not normalized. (PPTX) [file pone.0213364.s001.pptx]

## Slide 1
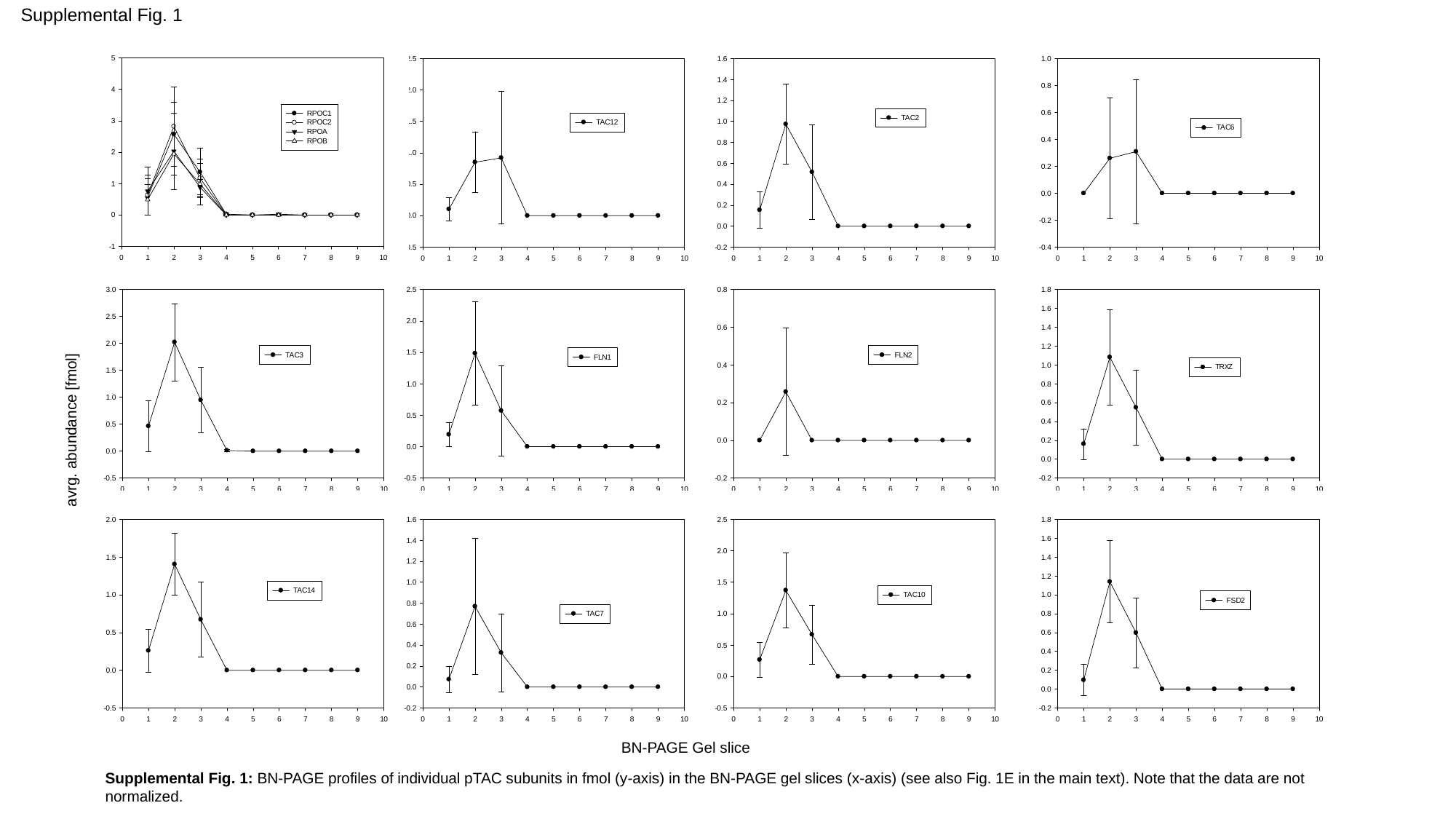

Supplemental Fig. 1
avrg. abundance [fmol]
BN-PAGE Gel slice
Supplemental Fig. 1: BN-PAGE profiles of individual pTAC subunits in fmol (y-axis) in the BN-PAGE gel slices (x-axis) (see also Fig. 1E in the main text). Note that the data are not normalized.
