# Supplementary figures and images for: Consequences of impaired 1-MDa TIC complex assembly for the abundance and composition of chloroplast high-molecular mass protein complexes
Source: PLoS One. 2019 Mar 13;14(3):e0213364. doi: 10.1371/journal.pone.0213364 (PMC6415892; doi:10.1371/journal.pone.0213364)

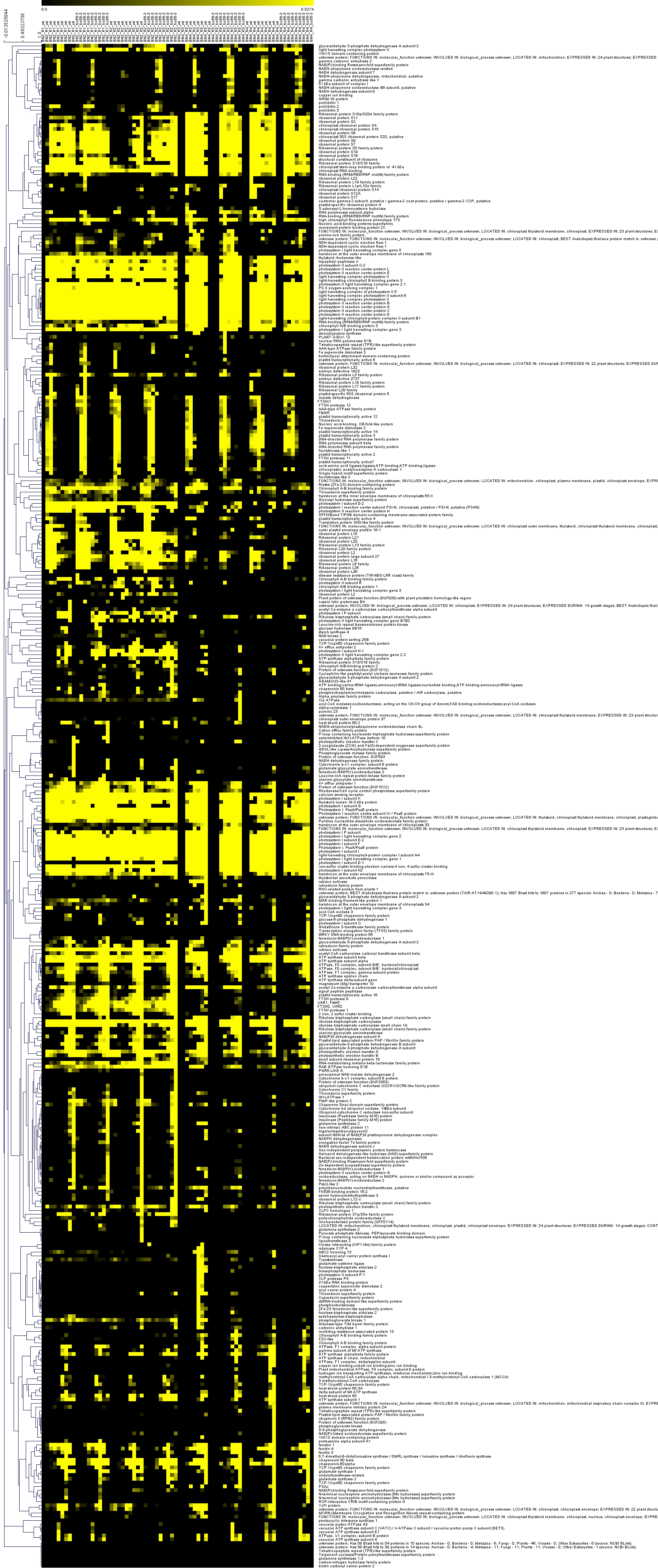

Supplement: S2 Fig — (TIF) [file pone.0213364.s002.tif]
