# Supplementary material for: Consequences of impaired 1-MDa TIC complex assembly for the abundance and composition of chloroplast high-molecular mass protein complexes
Source: PLoS One. 2019 Mar 13;14(3):e0213364. doi: 10.1371/journal.pone.0213364 (PMC6415892; doi:10.1371/journal.pone.0213364)
Supplement: S3 Fig — The BN-PAGE gel slice number is provided on the x-axis, the abundance (not normalized, in fmol) is provided on the y-axis. (PPTX) [file pone.0213364.s003.pptx]

## Slide 1
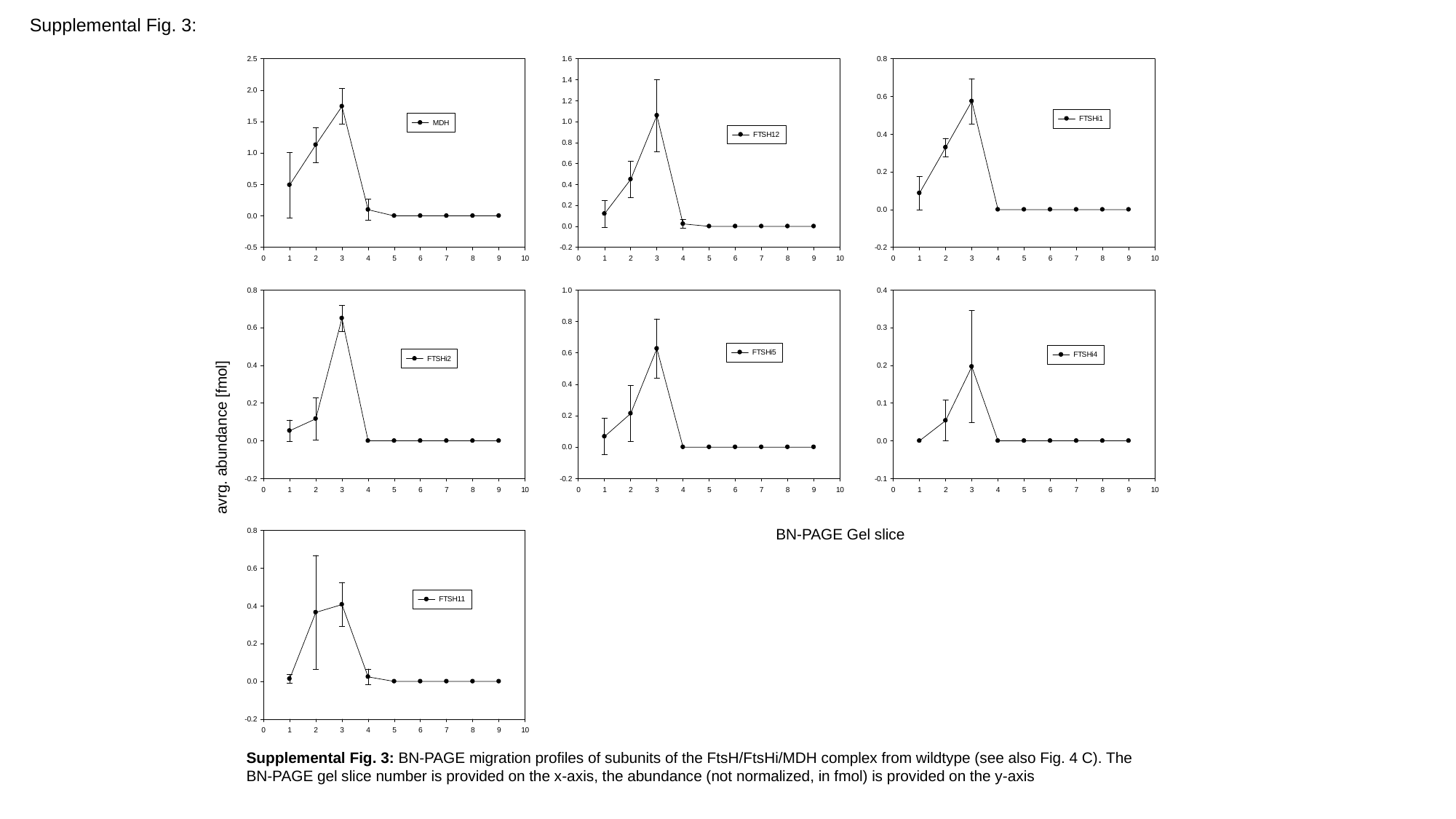

Supplemental Fig. 3:
avrg. abundance [fmol]
BN-PAGE Gel slice
Supplemental Fig. 3: BN-PAGE migration profiles of subunits of the FtsH/FtsHi/MDH complex from wildtype (see also Fig. 4 C). The BN-PAGE gel slice number is provided on the x-axis, the abundance (not normalized, in fmol) is provided on the y-axis
